# School and area-level disparities in exclusions in Scottish secondary schools

**DOI:** 10.23889/ijpds.v8i2.2194

**Published:** 2023-09-14

**Authors:** Morag Treanor, Patricio Troncoso, Lee Williamson

**Affiliations:** 1 University of Glasgow, Glasgow, United Kingdom; 2 University of Edinburgh, Edinburgh, United Kingdom

## Objectives

This paper explores the patterning of educational exclusions in Scottish secondary schools by variation across schools and council areas, and by structural socioeconomic factors and demographic characteristics of the pupils, their families, their schools and the areas in which they reside.

## Method

This research uses the newly linked administrative database created under the “Children’s Lives and Outcomes” research strand of the Scottish Centre for Administrative Data Research (SCADR). This linkage, the first of its kind in Scotland, includes data from Education Analytical Services and the Information Services Division of NHS Public Health Scotland from the period between 2007-2019, and the 2001 and 2011 Census. We adopt a Multilevel Modelling approach to ascertain the extent of the variation in the likelihood of a student being excluded across schools and council areas and its association with individual, school and area-level characteristics.

## Results

Preliminary results suggest that the variation in exclusions across secondary schools in Scotland is substantial and significant in terms of size and importance. Moreover, variation across council areas is also non-negligible, and is smaller than the variation found between-schools. This suggests that the effect of policy and/or practice at the school level is greater than that at the local authority level. Our analyses continue and are currently focusing on prior exclusions in primary school, deprivation, mental health, household and demographic characteristics, as well as school and area-level indicators. We expect to be able to elucidate further the relationships and interrelationships between schools, areas and family circumstances in the likelihood of being excluded from school.

## Conclusion

Our findings are pertinent to policymakers and practitioners in the context of a widening socio-economic gap exacerbated by COVID-19 restrictions and the current economic turmoil, to reduce the inequalities in exclusions and ultimately improve school experiences and outcomes.

